# Effects of exposure to sexually explicit material on sexually violent behavior among first-year university men in Vietnam

**DOI:** 10.1371/journal.pone.0275246

**Published:** 2022-09-27

**Authors:** Irina Bergenfeld, Yuk Fai Cheong, Tran Hung Minh, Quach Thu Trang, Kathryn M. Yount

**Affiliations:** 1 Department of Global Health, Rollins School of Public Health, Emory University, Atlanta, Georgia, United States of America; 2 Department of Psychology, Emory University, Atlanta, Georgia, United States of America; 3 Center for Creative Initiatives in Health and Population, Cầu Giấy, Hanoi, Vietnam; SUNY Downstate Health Sciences University, UNITED STATES

## Abstract

**Background:**

Adolescence and emerging adulthood represent a period of heightened vulnerability to sexual violence (SV). While some research suggests that exposure to sexually explicit material (SEM) among adolescents and college students is associated with sexually violent behavior, our understanding of this relationship is limited. This study aimed to assess the relationship between prior exposure to several types of SEM and sexually violent behavior in a sample of first-year university men in Vietnam.

**Methods and findings:**

A cohort of 739 first-year male university students completed three survey waves over 14 months, providing information on contact and non-contact sexually violent behavior, exposure to SEM, and other theorized confounders of the SEM-SV relationship. Controlling for these covariates, we estimated the average treatment effect of SEM on contact and non-contact SV using the propensity score method. We also conducted a dose-response analysis for the effect of violent SEM on SV based on frequency-of-exposure classes derived from latent class analysis. The majority of the sample reported exposure to SEM in the prior six months, with 41% of the sample reporting exposure to violent SEM. In propensity-adjusted models, exposure to violent SEM, but not other types, had a small but significant positive effect on contact and non-contact SV. These effects increased for frequent viewers of violent SEM. Models of contact SV showed endogeneity, warranting caution.

**Conclusions:**

Exposure to violent SEM is prevalent among university men in Vietnam and may be contributing to sexually violent behavior. Incorporating media literacy into SV prevention programs to mitigate these potential effects may be warranted.

## Introduction

Sexual violence (SV)—sexual acts committed against a person without freely given consent—is a global public health and human rights problem [1]. Late adolescence/emerging adulthood, especially the transition to university, is a period of heightened risk for SV [2], with about one in five college women in the US experiencing a campus sexual assault [3] and almost 9 in 10 male perpetrators of SV completing their first assault by age 20 [4]. Although less is known about the rates of SV on college campuses in low- and middle-income countries (LMICs), estimates from large multi-country studies confirm that, in general, men’s reported sexually violent behavior and women’s reported victimization are high [1,5].

In a small number of studies among adolescents and emerging adults, exposure to sexually explicit material (SEM) has been positively associated with the risk of perpetrating SV [6–9], with substantive differences based on the medium [8] and content [6] of exposure. However, our understanding of this relationship remains limited [10,11], especially in LMICs. The potential for reciprocity between SEM and SV also warrants caution when interpreting causality within the SEM-SV relationship [9]. As access to the internet continues to expand globally, SEM is becoming more widely available to adolescents [12,13]. Therefore, the need is increasing to understand how young men’s exposure to such content may influence their risk of sexually violent behavior, and which types of content may contribute to this effect.

## Background

### Exposure to sexually explicit material among adolescents

SEM, often used interchangeably with “pornography,” is media which depicts intercourse, sexual contact, or sexual objectification, including “domination, degradation, subordination, or humiliation of women” [11]. Prevalence estimates of adolescent exposure to SEM vary greatly—from as low as 7% to as high as 98%—depending on the types of media considered; the age, gender, and location of the sample; the window of exposure; whether exposure is intentional; and the methods used [10]. Nevertheless, expanding access to the internet and to mobile phones in the past decade, especially in LMICs, has made SEM more accessible at younger ages [12,13].

Several studies have examined individual and contextual factors that may predict intentional use of SEM [10,14]. Diverse studies conducted in several countries have shown that boys are more likely to intentionally use SEM than girls [10], although gender differences are minimal in more socially liberal countries [15]. According to two large studies in European countries, male adolescents who identified as homosexual or bisexual were more likely than their heterosexual peers to access online SEM [16,17], and mixed-methods research suggests that online SEM may be used as an important resource for sex education among sexual minority youth [18]. In studies among adolescents in Europe [16,19] and the US [7], sensation-seeking was associated consistently with more frequent exposure to SEM for both girls and boys. One large longitudinal study among Dutch adolescents linked hyper-gender ideology with increased use of SEM featuring violence in both boys and girls [19]. While studies have assessed the relationship between religiosity and use of SEM across a wider range of contexts, findings were mixed, including negative and null associations [10,14]. Studies on the impacts of age and sexual experience on use of SEM reflect similarly inconsistent findings [14], though evidence is stronger that more advanced pubertal stage is positively associated with more frequent SEM use [10]. Broadly, adolescents who “violated societal norms” were more frequent users of SEM; however, “norms violation” was defined variously across studies [10]. At the contextual level, family dynamics, including coercive parental discipline [20], poor family functioning [21], poor caregiver bonding [20], and less caregiver monitoring [20] were associated with SEM use in studies conducted in the US and Hong Kong.

### Influence of exposure to sexually explicit material on sexually violent behavior

Overall, the literature linking exposure to SEM and SV behavior among adolescents and emerging adults is sparse. Firstly, the large majority of studies are cross-sectional and conducted in the US, limiting researchers’ ability to draw causal, generalizable conclusions [10,22]. Secondly, findings from the few rigorous, longitudinal studies examining the relationship between SEM exposure and sexually violent behavior among adolescents or emerging adults were mixed and dependent on the type and content of media consumed [10,22]. To date, three longitudinal studies conducted among young people in the US have found a positive association between SEM exposure and sexually violent behavior [6,7,9], including sexual harassment [7,9]. One study among 10–16 year-olds found that both boys and girls accessing violent SEM had six times the odds of perpetrating acts of sexual aggression than those who did not access violent SEM [6]. However, the same study found that SEM use in general was not associated with increased likelihood of sexual aggression [6], complementing research among college men suggesting that violent SEM is associated with rape proclivity and actual rape, while non-violent SEM shows no association with rape proclivity or actual SV [23]. A 2009 study found that US middle school students’ exposure to any SEM was associated with subsequent sexual harassment behavior for boys, but not girls [7]. On the other hand, a recent longitudinal study of Croatian high school boys found no link between exposure to SEM and sexual aggression after controlling for personality traits including bullying, delinquency, callousness, impulsiveness, and peer pressure; however, this study used a single indicator to measure SEM exposure and did not consider the type of content viewed [24]. Finally, studies conducted among college men suggest that SEM may have unique effects on young men who are at higher risk for sexual aggression but negligible effects on men considered to have lower rape proclivity [25,26].

Media-studies researchers have proposed several theoretical models to explain the impact of SEM on sexually violent behavior through alterations in attitudes, beliefs, and perceptions [11]. Prominent among these are cultivation theory, which posits that media consumption affects the viewer’s perceptions and expectations about the real world [27], and social-cognitive theory, which suggests that human behavior is influenced by multilevel factors including self-efficacy, outcome expectations, and perceived environmental barriers and facilitators [28]. Thus, several studies have examined the impact of SEM exposure on attitudes, such as rape myth acceptance [29–31], beliefs about gender roles [7], self-reported masculinity [32], and adversarial sexual beliefs [30,31] to explain the potential impact of SEM on behavior. Uses-and-gratifications theory, which views media effects through the lens of individual desires and needs [33], also has been used to frame individual differences in propensity to access SEM [26,29], suggesting a more reciprocal relationship between SEM use and sexually violent behavior. A 2022 study conducted among US middle and high school students supports this theory, finding that sexual harassment perpetration predicted subsequent SEM use among both girls and boys, using a cross-lagged design [9].

### Study aims

To date, few studies of SEM exposure and its relationship with sexually violent behavior have been conducted outside the US, and none in LMICs [10]. Moreover, studies demonstrating an association between sexually violent behavior and SEM exposure among adolescents have not incorporated propensity scores to control for the possibility that individuals likely to engage in such behavior also may be likely to seek out SEM [9], raising questions regarding causality. The present study aimed to assess the relationship between prior exposure to different types of SEM, whether intentional or unintentional, and sexually violent behavior in a sample of men attending their first year of university in Vietnam, controlling for observed theorized predictors of SEM exposure and sexually violent behavior.

## Methods

This analysis used data from the randomized controlled trial of *GlobalConsent* (NCT04147455), a web-based program designed to prevent sexually violent behavior among university-going men [34]. The Institutional Review Boards at Emory University (IRB00099860) and the Hanoi University of Public Health (017–384/DD-YTCC) approved the study. The study sample size of 800 was calculated using a Monte Carlo approach [35] in MPlus (Version 8.0, Muthen and Muthen, San Francisco, California) considering small to large effect sizes [36], with a power of 0.80, and accounting for 10% predicted attrition [34]. This study found that assignment to *GlobalConsent*, versus an attention-control program, prevented increases in sexually violent behavior in the 10–13 months following the program, decreasing the odds of engaging in any act of sexually violent behavior by 29% and the odds of two or more acts by 53% [37].

### Setting

Our study was conducted at two universities in Hanoi, Vietnam. University 1 is a public university specializing in the health professions and enrolling 1000 students annually, 40% of whom are men. University 2, a private university, trains students in diverse disciplines and enrolls 7000 students annually, 45%-50% of whom are men.

### Measures

For all measures used in data collection, we adapted existing validated scales and translated them into Vietnamese where no existing translation was available [34]. All scales were back-translated into English to ensure accuracy and underwent cognitive testing among eight male college students and refinement before administration.

### Outcomes

We used a modified Sexual Experiences Survey (SES) [38] to capture sexually violent behaviors in the prior six months at all three survey waves. While this tool has not, to the authors’ knowledge, been validated in Vietnam prior to the *GlobalConsent* study, it has been validated in various populations [39,40], including among Asian-Americans [41,42]. Psychometric testing as part of the parent study confirmed the validity of the SES in the sample (Supplemental Materials). Participants indicated the frequency (never, once, twice, three or more times) with which they had perpetrated each of 10 acts of non-contact SV derived from the longform SES. Participants also selected the frequency (never, once, twice, three or more times) with which they had used each of five tactics (e.g. coercion, physical force, deception) to derive from the shortform SES perpetrate seven acts of contact SV. An example of non-contact SV was, “I sent someone sexual or obscene materials, such as pictures, jokes, or stories in the mail or over the Internet, after they had asked me to stop.” An example of contact SV was, “I fondled, kissed, or rubbed up against the private areas of someone’s body (lips, breast/chest, crotch or butt) or removed some of their clothes when they did not agree to it (but did not attempt sexual penetration).” Acts of non-contact SV and contact SV each were combined into single, dichotomous variables: any (one or more acts) versus none. Contact sexually violent behavior was refined further into two dichotomous variables representing any acts (yes/no) perpetrated by physical means, including violence or threat of violence, and any acts (yes/no) perpetrated by non-physical means, including deception, verbal coercion, or intoxication.

### Exposures

Using a tool adapted from the Kids Online survey [43], participants were asked how often they were exposed to five types of SEM (text-based, partial nudity, full nudity, sexual acts, and violent sexual acts) through various media (internet browser, TV/movie/DVD, book/magazine, or social media) in the prior six months. We recoded these responses into a single, four-level categorical variable: no exposure to SEM (0), exposure to text-based SEM or nudity only (1), exposure to non-violent sexual acts (2), and exposure to violent sexual acts (3). For the present study, we defined SEM involving violent sexual acts as “images or video of a woman performing a sex act in which she was choked, hit, humiliated, or forced.” From the multi-level variable, we also created three dummy variables: exposure to any SEM (0 vs. 1/2/3), exposure to any sexual acts (0/1 vs. 2/3), and exposure to violent sexual acts (0/1/2 vs. 3).

### Covariates

Covariates were selected for our models based on conceptual associations with the outcome of sexually violent behavior. Demographic characteristics collected at baseline included age, sexual orientation (heterosexual vs. bisexual), religiosity (any religion vs. none), living situation (with parents vs. others), relationship history (ever vs. never in a relationship), and ethnicity (Kinh majority vs. minority). Covariates also included information measured at endline on childhood maltreatment, defined as acts of abuse or neglect by adults the respondent experienced before age 18 [44]. We measured childhood maltreatment using a scale adapted and validated in Vietnam and summed responses to its 27 dichotomous items to create a single maltreatment score [44]. Finally, we calculated propensity scores for each SEM exposure variable (ordinal and dichotomous) using covariates theorized to be associated with sexually violent behavior based on prior literature [45]: ethnicity as a proxy for socioeconomic status [46], childhood maltreatment [47], living situation as a proxy for parental oversight [20], relationship history, and religiosity [48], as well as baseline outcomes, university, and trial arm. Although the *GlobalConsent* program did not explicitly address SEM, it did include content about rape myths, which could potentially mediate the relationship between SEM and sexually violent behavior [25]; thus, trial arm was included in the propensity score to account for possible effects of the intervention of men’s propensity to view SEM. This allowed us to use the full sample of participants while controlling for any potential bias introduced by exposure to the intervention.

#### Eligibility and recruitment

From the list of students who matriculated at the two universities in September 2019, all male students from departments with >15 male students (University 1) or >20 male students (University 2) were invited to participate in the study. A total of 362 in University 1 and 735 in University 2 were invited to attend orientation meetings for students in September 2019 at the two universities to introduce the intervention and the study. All male students ages 18 to 24 years who attended the orientation meetings were invited to participate in the baseline survey and *GlobalConsent* program. Sixteen students from University 1 and 189 students from University 2 did not attend, and seven students from University 2 refused to participate following the meeting. In total, 345 men from University 1 and 457 men from University 2 finished the baseline survey.

#### Data collection

In September 2019, written informed consent was emailed to participants in advance and was signed in person at the study sites before taking part in the baseline survey (wave 1) [49]. A total of 802 participants self-administered a survey using tablets installed with REDCap mobile app software, and participants were compensated 100000 VND (about 4.00 USD). Nine of 802 participants were deemed ineligible after completing the first survey, bringing the baseline sample to 793. Due to COVID-19 restrictions, participants completed the two planned follow-up surveys online in March-April of 2020 (wave 2, n = 751, 95% retention) and October-November 2020 (wave 3, n = 739, 93% retention). For these, compensation was increased to 150000 VND (about 6.50 USD) to ensure retention without coercion, and consent was collected electronically. On average, each survey wave took 45 minutes to complete.

#### Analysis

**Fig 1:**
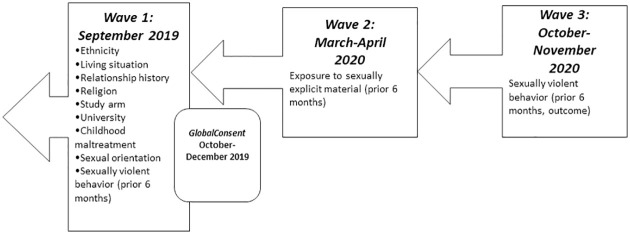
Three-wave longitudinal analysis.

First, we assessed the characteristics of the sample with descriptive statistics, overall and across the four SEM exposure groups for all 739 participants, using baseline demographic characteristics at wave 1, SEM exposure at wave 2, and sexually violent behavior at wave 3 (Fig 1).

Four participants missing ethnicity data were excluded from later analyses via listwise deletion. Second, we regressed all SV outcomes measured at wave 3 on the dichotomous SEM exposures measured at wave 2, unadjusted and adjusted for theorized confounders, including religion, ethnicity, sexual orientation, university, trial arm, childhood maltreatment, baseline relationship history, baseline living situation, and baseline sexually violent behavior. Third, we created propensity scores [45] to estimate the conditional probability of SEM exposure (any, sexual acts, and violent sexual acts) given the above covariates and calculated the average treatment effect (ATE) on sexually violent behavior using inverse probability-weighted regression adjustment (IPWRA), a “doubly robust” method for reducing bias in observational studies [50]. Age was excluded from the propensity score and outcome models due to low variability, as the overwhelming majority of the sample was 18 years old. To test for balance across categories of SEM exposure, which provides greater support for causal inference [45], we visually assessed the overlap of plots of propensity scores across each dichotomous SEM exposure variable and across the four-level categorical variable. A greater degree of overlap suggests balance across propensity groups, or a greater likelihood that individuals not “treated” also have a probability of being “treated” and vice versa, lending greater support for causal inference [51]. We also conducted chi-square over-identification tests on the dichotomous SEM exposure variables using the *teffects* function [50]. A nonsignificant p-value for the overidentification test fails to reject the null hypothesis that covariates are balanced across exposure groups, enabling causal inference [50]. Finally, we tested for endogeneity, or bias due to unmeasured covariates associated with “treatment” (dichotomous SEM exposure variables) and SV outcomes, using the *eteffects* module [50]. A significant endogeneity test suggests the presence of unmeasured confounding in the model [50]. These analyses were conducted using STATA (Release 16, StataCorp LP, College Station, Texas). We performed a sensitivity analysis using only the control arm to reduce further any bias from exposure to the *GlobalConsent* intervention.

We also performed a supplemental analysis to assess the dose-response relationship of exposure to violent SEM with sexually violent behavior. Specifically, we modeled latent classes of exposure to violent SEM using four indicator variables: frequency of exposure via print media, via web, via television/movie, and via social media. Each indicator variable had five response options: never, less than once a month, one to three times monthly, one to three times weekly, and daily or almost daily. We used mixture models in MPlus (Version 8.0, Muthen and Muthen, San Francisco, California) to estimate solutions for two, three, four, and five classes of exposure frequency. We selected a final model based on model fit statistics, including log likelihood, Aikake Information Criterion (AIC), Bayesian Information Criterion (BIC), as well as homogeneity, discrimination, entropy, and considerations of parsimony and theoretical coherence [52]. We exported a class indicator variable into STATA (Release 16, StataCorp LP, College Station, Texas) to perform unadjusted and adjusted regressions of each SV outcome on dummy variables representing the cutpoints of exposure-frequency class: frequent/infrequent vs. none and frequent vs. infrequent/none. Finally, we created propensity weights to calculate ATE with IPWRA for each SV outcome.

## Results

### Characteristics of the sample

On average, participants were 18 years old, and had experienced between three and four types of childhood maltreatment (Table 1). Participants were split roughly equally between the two universities and between treatment and attention-control arms. More than one third lived with their parents. Roughly 95% of our sample self-identified as ethnically Kinh, versus 86% in Vietnam as a whole. Less than 5% of the sample identified as bisexual, and roughly 17% identified as religious (Buddhist, Christian, or other). Just under half of participants had ever been in a sexual or romantic relationship at baseline. Most participants (86%) reported exposure to any type of SEM, including text-based, nudity, and sexual acts. Nearly two thirds of participants reported exposure to SEM involving sex acts, and of these, 62% had viewed violent sex acts (41% of the total sample). The score for childhood maltreatment was positively associated with the level of SEM exposure, and the percentage of those reporting sexually violent behavior in the six months before baseline was at least twice a high for those exposed to violent SEM versus other exposure categories.

**Table 1:** Characteristics of first year university men in Hanoi, Vietnam by SEM exposure level (n = 739).

	Full sample (n = 739)	No SEM exposure at post-test 1 (Level 0) (n = 102)	Exposure to text-based SEM and/or nudity only (Level 1) (n = 149)	Exposure to any SEM with non-violent sexual acts (Level 2) (n = 185)	Exposure to any SEM with violent sexual acts (Level 3) (n = 303)
	Mean (SD) or Percent	Mean (SD) or Percent	Mean (SD) or Percent	Mean (SD) or Percent	Mean (SD) or Percent
Age	18.06 (0.39)	18.07 (0.45)	18.05 (0.30)	18.04 (0.22)	18.09 (0.48)
With parents	30.45%	30.39%	32.21%	31.89%	28.71%
On campus	15.97%	16.67%	23.49%	12.43%	14.19%
Others	53.59%	52.94%	44.30%	55.68%	57.10%
Sexual orientation (bisexual vs. heterosexual)	4.47%	2.94%	4.70%	4.86%	4.62%
Religion (any vs. none)	17.32%	18.63%	13.42%	15.14%	20.13%
Relationship history (ever vs. never)	45.47%	35.29%	42.28%	46.49%	49.83%
Ethnicity (minority vs. majority) [4 missing]	4.35%	5.00%	4.70%	1.62%	5.65%
Childhood maltreatment (summative score)	3.78 (4.95)	2.22 (4.09)	3.20 (3.86)	4.11 (4.34)	4.40 (5.84)
Treatment arm (GlobalConsent vs. AHEAD)	49.26%	50.00%	53.02%	50.81%	46.20%
University (1 vs. 2)	46.14%	49.02%	51.01%	49.19%	40.92%
Any non-contact SV (baseline)	23.68%	8.82%	16.78%	17.84%	35.64%
Any contact SV (baseline)	12.72%	7.84%	7.38%	7.57%	20.13%
Contact SV via physical tactics (baseline)	6.50%	5.88%	4.03%	3.24%	9.90%
Contact SV via non-physical tactics (baseline)	11.23%	4.90%	6.71%	7.03%	18.15%
Any non-contact SV (wave 3)	16.24%	6.86%	10.07%	7.57%	27.72%
Any contact SV (wave 3)	16.51%	5.88%	12.75%	9.73%	26.07%
Contact SV via physical tactics (wave 3)	9.47%	3.92%	6.71%	5.41%	15.18%
Contact SV via non-physical tactics (wave 3)	15.56%	5.88%	10.74%	9.73%	24.75%

### Effect of sexually explicit material exposure on sexual violence

**Fig 2:**
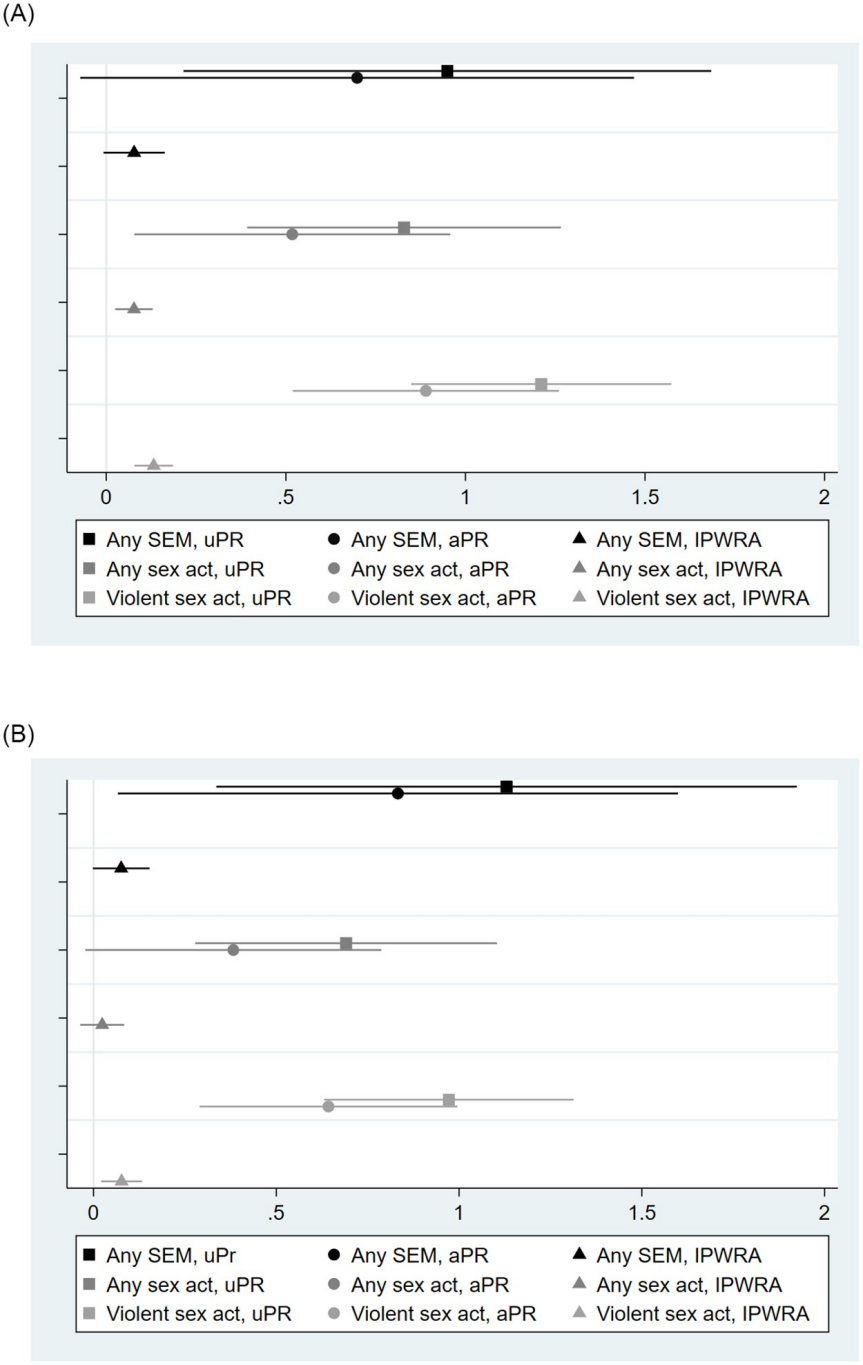
Effect of exposure to sexually explicit material on non-contact and contact sexually violent behavior among first-year university men in Hanoi, Vietnam (n = 735).

In unadjusted poisson regressions, exposure to any SEM, exposure to SEM with sexual acts, and exposure to SEM with violent sexual acts all were associated with about two to three times the prevalence of sexually violent behavior in the prior six months (Table 2 & Fig 2). After adjusting for covariates, all prevalence ratios were substantially attenuated, remaining consistently significant only for exposure to violent SEM. Covariate-adjusted prevalence ratios also remained significant for the models of any SEM with contact SV, any sexual acts with non-contact SV, and any sexual acts with contact SV via non-physical tactics. In models incorporating propensity scores and adjusting for all covariates, exposure to SEM with any sexual acts and violent sexual acts predicted small but significant increased prevalence of non-contact SV (8% and 14%, respectively). Finally, exposure to violent SEM remained the only significant predictor of contact SV and contact SV via non-physical tactics in models estimating the ATE with IPWRA, although effect sizes were much smaller than those obtained using regression adjustment only, with increased prevalence of 9% and 4%, respectively. No exposures were significantly associated with the prevalence of contact SV via physical tactics after IPWRA. Sensitivity analysis restricting the sample to the attention-control group only produced similar results (S1 Table), as did the use of a four-level categorical exposure variable (S2 Table), with violent SEM exposure being the only consistently significant predictor of sexually violent behavior after IPWRA.

**Table 2:** Effect of exposure to sexually explicit material on sexually violent behavior among first-year university men in Hanoi, Vietnam (n = 735).

Outcome	uPR	95% CI	aPR	95% CI	ATE with IPWRA	95% CI
Non-contact SV						
0 vs. 1/2/3	2.58*	[1.24–5.39]	2.01	[0.93–4.35]	1.08	[0.99–1.18]
0/1 vs. 2/3	2.29**	[1.48–3.55]	1.68*	[1.08–2.61]	1.08**	[1.03–1.14]
0/1/2 vs. 3	3.36**	[2.34–4.82]	2.43**	[1.68–3.53]	1.14**	[1.08–1.20]
0 vs. 1/2/3	3.10**	[1.40–6.85]	2.30*	[1.07–4.95]	1.08	[1.00–1.17]
0/1 vs. 2/3	2.00**	[1.32–3.02]	1.47	[0.98–2.20]	1.02	[0.96–1.09]
0/1/2 vs. 3	2.64**	[1.88–3.72]	1.90**	[1.34–2.71]	1.09**	[1.03–1.14]
Contact SV: physical tactics						
0 vs. 1/2/3	2.64	[0.98–7.10]	1.90	[0.71–5.07]	1.00	[0.81–1.23]
0/1 vs. 2/3	2.06*	[1.17–3.62]	1.47	[0.83–2.60]	1.00	[0.94–1.06]
0/1/2 vs. 3	2.76**	[1.72–4.42]	1.98**	[1.21–3.26]	1.04	[0.98–1.09]
Contact SV: non-physical tactics						
0 vs. 1/2/3	2.91**	[1.31–6.44]	2.07	[0.96–4.46]	1.07	[0.99–1.15]
0/1 vs. 2/3	2.17**	[1.40–3.37]	1.57*	[1.02–2.40]	1.03	[0.97–1.09]
0/1/2 vs. 3	2.70**	[1.89–3.85]	1.89**	[1.32–2.72]	1.07*	[1.03–1.13]

*Significant at <0.05;

**Significant at <0.01;

uPR = unadjusted prevalence ratio; aPR = adjusted prevalence ratio; ATE with IPRWA = average treatment effect with inverse probability weighted regression adjustment; df = degrees of freedom. Four participants missing covariates were excluded. Exposure categories were as follows: 0 = no exposure; 1 = nudity or text only; 2 = SEM with sexual acts; 3 = SEM with violent sexual acts.

Together, overlap plots and over-identification tests (S3 Table) suggested balanced propensity scores across all three dichotomous SEM exposures. Endogeneity tests for non-contact SV were non-significant, suggesting that unmeasured confounding was trivial. Some models estimating contact SV outcomes, however, showed evidence of significant, residual unmeasured confounding.

### Latent classes for exposure to violent SEM

The most parsimonious and theoretically coherent latent-class model for the frequency of exposure to violent SEM was a three-class solution (S4 Table). Our final model included a little-to-no-exposure class (>0.99 conditional probability of choosing “never” for each item) representing 63% of participants, an infrequent-exposure class (>0.81 conditional probability of choosing “less than once a month” for each item) representing 20% of participants, and a frequent-exposure class (>0.98 conditional probability of choosing “on to three times monthly” or greater on each item) representing 17% of participants.

### Dose-response effects for latent-classes of exposure to violent SEM

**Fig 3:**
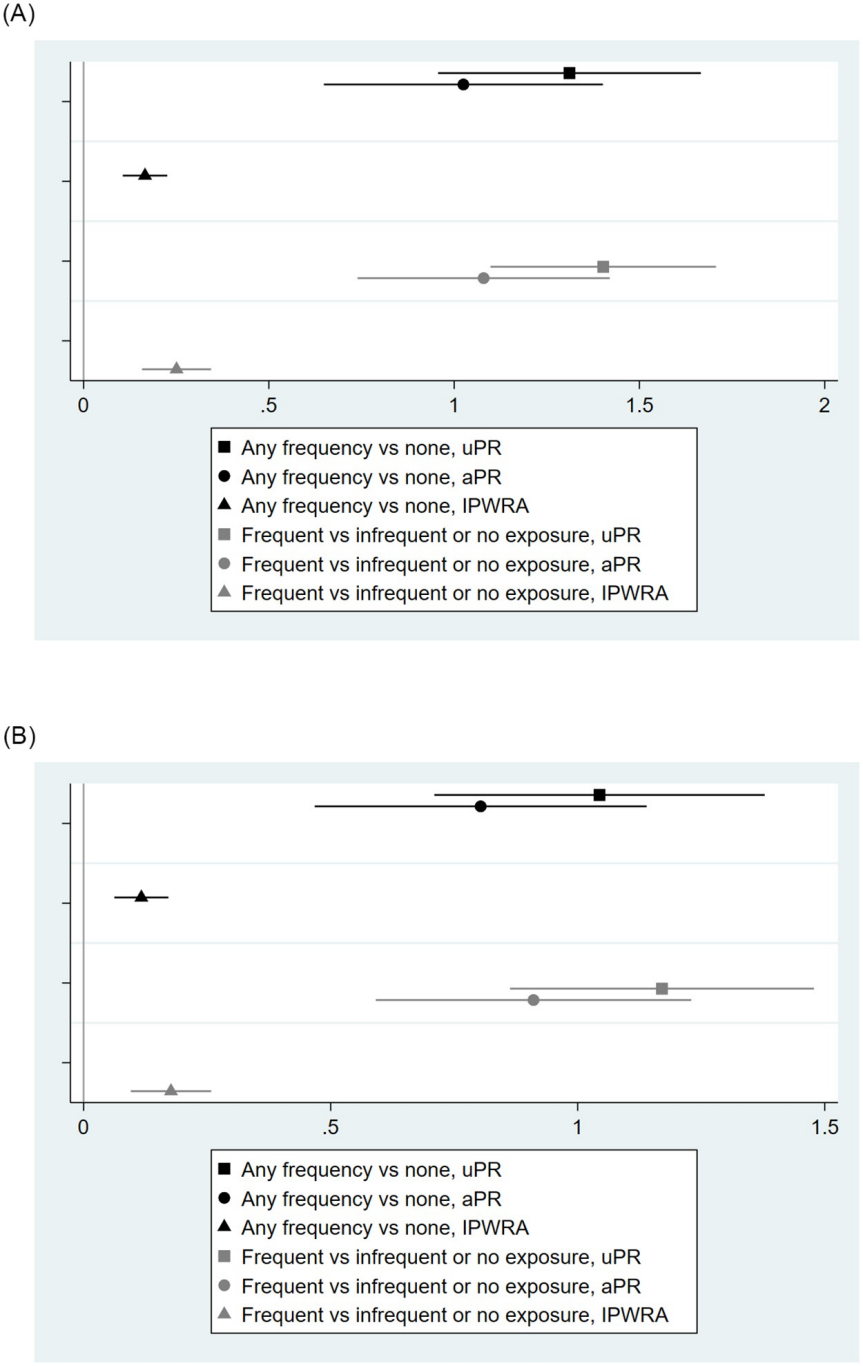
Effect of violent sexually explicit material exposure class on non-contact and contact sexually violent behavior among first-year university men in Hanoi, Vietnam (n = 733).

In unadjusted poisson regression, those in the frequent or infrequent violent SEM exposure class had about three times the prevalence of sexually violent behavior in the prior 6 months versus the no exposure class (Table 3 & Fig 3). For members of the frequent exposure class, prevalence of sexually violent behavior was three to five times as great as infrequent- or no-exposure classes. In models adjusted for covariates, all prevalence ratios decreased in magnitude but remained significant. After IPWRA, infrequent or frequent class membership resulted in 18% higher prevalence of non-contact acts and 12% higher odds of contact acts, both significant at p<0.01. For the frequent exposure class versus other classes, these increased prevalences were 29% and 19%, respectively. Estimates for contact SV through physical vs. non-physical tactics did not differ substantively.

**Table 3:** Effect of violent sexually explicit material exposure class on sexually violent behavior among first-year university men in Hanoi, Vietnam (n = 733).

Outcome	uPR	95% CI	aPR	95% CI	ATE with IPWRA	95% CI
Non-contact SV						
Class 2/3 vs. 1	3.71**	[2.60–5.29]	2.79**	[1.91–4.06]	1.18**	[1.11–1.25]
Class 3 vs. 1/2	4.06**	[3.00–5.51]	2.94**	[2.09–4.14]	1.29**	[1.17–1.41]
Any contact SV						
Class 2/3 vs. 1	2.84**	[2.03–3.97]	2.23**	[1.60–3.13]	1.12**	[1.06–1.19]
Class 3 vs. 1/2	3.22**	[2.37–4.39]	2.49**	[1.81–3.42]	1.19**	[1.10–1.30]
Contact SV: physical tactics						
Class 2/3 vs. 1	3.75**	[2.32–6.08]	2.79**	[1.69–4.62]	1.09**	[1.04–1.14]
Class 3 vs. 1/2	5.09**	[3.31–7.80]	3.82**	[2.39–6.12]	1.17**	[1.09–1.26]
Contact SV: non-physical tactics						
Class 2/3 vs. 1	2.77**	[1.96–3.92]	2.10**	[1.48–2.98]	1.10**	[1.05–1.16]
Class 3 vs. 1/2	3.20**	[2.32–4.41]	2.39**	[1.71–3.33]	1.17**	[1.08–1.26]

*Significant at <0.05;

**Significant at <0.01;

uPR = unadjusted prevalence ratio; aPR = adjusted prevalence ratio; ATE with IPRWA = average treatment effect with inverse probability weighted regression adjustment; df = degrees of freedom. Four participants missing covariates were excluded. Frequency classes are as follows: 1 = unexposed to violent SEM; 2 = exposed less than monthly; 3 = exposed ≥1 time per month.

Based on overlap plots and overidentification tests, balance in propensity scores was achieved across exposure classes (S5 Table). Endogeneity tests revealed that unmeasured confounding was minimized in models of non-contact SV, but remained present in some models of contact SV, particularly via non-physical tactics.

## Discussion

This longitudinal analysis of the relationship between exposure to SEM and sexually violent behavior among university students in Vietnam contributes to a nascent body of research that, to date, has been mainly cross-sectional and conducted among adolescents and emerging adults in the US and Europe. The overwhelming majority of young men in our sample reported exposure to SEM at least once in the prior six months, corroborating findings from another recent study in Hanoi [53]. In our study, more than half of those exposed to SEM had viewed violent content. Exposure to violent SEM, but not other types of SEM, was associated with a small but consistent increase in the prevalence of sexually violent behavior, an effect that increased with the dose of exposure to violent SEM.

### Limitations and strengths of the study

This analysis was conducted using data from two universities in Hanoi and may not be generalizable to other populations in Vietnam, including university women and youth attending universities in other regions of Vietnam. Replication of our findings among these groups will be needed to determine whether our results are more broadly applicable. Furthermore, while we purposefully did not distinguish between intentional and unintentional exposure to SEM, there may be key differences in the effect of SEM depending on viewer’s intent, which will be explored in future analyses. Also, the significant results of some of the endogeneity tests suggested that unobservable confounding continued to affect the SEM-contact SV relationship, specifically. Unmeasured confounding may have resulted from school closures and lockdowns during the COVID-19 pandemic, which coincided with our study period and may have caused some variability in students’ living situation and internet access and limited their contact with peers, decreasing opportunities for physical contact.

For ethical and practical reasons, it is not possible to randomize SEM exposure in studies of its effects on violent sexual behavior. Prior cross-sectional, observational studies have employed regression-based approaches to estimate the association between exposure to SEM and such behavior in adolescent populations, finding very large effect sizes for exposure to violent SEM [6]. We used propensity scores with longitudinal data to balance the distribution of observed characteristics across exposure groups [45], which might predispose certain individuals to seek out SEM. Compared to regression adjustment, incorporating propensity scores resulted in considerably smaller, but still significant, effect sizes, suggesting that regression-based methods alone are insufficient to reduce bias in studies of SEM exposure and sexually violent behavior.

### Implications for research and practice

Findings from this study have implications for SEM research in adolescent and emerging adult populations. First, it is important to measure not only the frequency, but the type of content in SEM exposure. Conflating violent and non-violent SEM exposure may mask significant effects and inappropriately attribute sexually violent behavior to forms of SEM that do not depict violent sexual acts. Conversely, conflating violent and non-violent SEM may mask significant effects of violent content. Finally, observational studies that do not account for individual differences in the propensity to access SEM run the risk of over-estimating effect sizes. Propensity score methods have the potential to produce more accurate estimates of the effect of SEM on sexually violent behavior than regression adjustment alone, even when autoregressive methods are used to control for prior values of the criterion variable [10].

Our findings also have important implications for efforts to reduce sexually violent behavior among university-going men in Vietnam, and perhaps elsewhere. First, men’s exposure to non-violent SEM has no significant effect on their risk of sexually violent behavior. If these findings are replicated, they suggest that such content does not appear to be harmful with respect to SV. Second, men’s exposure to violent SEM does appear to heighten their risk of sexually violent behavior, especially for non-contact SV and if SEM is viewed frequently. Interpretation of this finding warrants caution, however, because of the potential presence of endogeneity in some models of contact SV. Third, the sub-sample of men who frequently access violent SEM may require tailored intervention that addresses the underpinnings of their heightened risk of exposure as well as the knowledge, attitudinal, affective, and behavioral effects of heightened exposure itself [26]. While research on SEM education designed for adolescents is still an emerging field, promising results from recent pilot studies of educational interventions in the US and Europe suggest that such programming may positively impact knowledge, attitudes, and behavioral intentions [54,55]. Such interventions also may benefit the general population of adolescents and emerging adults in ways unrelated to reducing sexually violent behavior, given research demonstrating other negative outcomes of SEM exposure, including body-image issues [14,56] and lower sexual satisfaction [57].

### Conclusions

Exposure to violent sexually explicit material is prevalent among university men in Vietnam and may be contributing to their risk of sexually violent behavior. Further research is needed to identify the cognitive, attitudinal, and/or affective pathways by which SEM exposure may impact SV risk in these populations. Programs to reduce SV among high-risk adolescents and emerging adults should consider incorporating contextualized, evidence-based education about the potential harms of violent sexually explicit material.

## Supporting information

S1 Table Results of sensitivity analysis results using control arm only. (PDF)

S2 Table Supplementary analysis using four-level categorical SEM exposure with overlap plots. (PDF)

S3 Table Overidentification tests, overlap plots, and endogeneity tests for main analysis. (PDF)

S4 Table Fit statistics for two to five latent classes of exposure to violent sexually explicit material. (PDF)

S5 Table Overidentification tests, overlap plots, and endogeneity tests for dose-response analysis. (PDF)
