# Supplementary material for: Effects of exposure to sexually explicit material on sexually violent behavior among first-year university men in Vietnam
Source: PLoS One. 2022 Sep 27;17(9):e0275246. doi: 10.1371/journal.pone.0275246 (PMC9514651; doi:10.1371/journal.pone.0275246)
Supplement: S1 Table — (PDF) [file pone.0275246.s001.pdf]

S1 Table. Effect of exposure to sexually explicit material on sexually violent behavior among first-year university men in Hanoi, Vietnam, control group only (n=375)

**Outcome**	**uPR**	**95% CI**	**aPR**	**95% CI**	**ATE with IPWRA**	**95% CI**
Non-contact SV						
0 vs. 1/2/3	2.40	[0.91-6.32]	1.89	[0.75-4.79]	1.06	[0.94-1.20]
0/1 vs. 2/3	2.10*	[1.17-3.79]	1.55	[0.87-2.78]	1.08	[1.00-1.16]
0/1/2 vs. 3	2.93**	[1.80-4.76]	2.11**	[1.29-3.42]	1.12**	[1.04-1.21]
Contact SV						
0 vs. 1/2/3	2.01	[0.85-4.77]	1.67	[0.75-3.71]	0.73	[0.11-5.03]
0/1 vs. 2/3	1.87*	[1.08-3.23]	1.50	[0.89-2.52]	1.03	[0.94-1.13]
0/1/2 vs. 3	2.44**	[1.55-3.83]	1.83*	[1.15-2.94]	1.05	[0.95-1.17]
Contact SV: physical tactics						
0 vs. 1/2/3	1.89	[0.60-5.92]	1.57	[0.51-4.86]	NC	NC
0/1 vs. 2/3	1.59	[0.78-3.24]	1.29	[0.64-2.60]	0.98	[0.87-1.10]
0/1/2 vs. 3	2.08*	[1.72-4.42]	1.61	[0.83-3.12]	0.96	[0.82-1.12]
Contact SV: non-physical tactics						
0 vs. 1/2/3	1.86	[0.78-4.41]	1.49	[0.67-3.31]	0.73	[0.11-5.02]
0/1 vs. 2/3	2.06*	[1.14-3.72]	1.60	[0.92-2.78]	1.03	[0.92-1.15]
0/1/2 vs. 3	2.48**	[1.89-3.85]	1.77*	[1.08-2.89]	1.04	[0.93-1.16]

*Significant at <0.05; **Significant at <0.01; uPR=unadjusted prevalence ratio; aPR=adjusted prevalence ratio; ATE with IPRWA=average treatment effect with inverse probability weighted regression adjustment. NC=no convergence.

S1 Table. Effect of violent sexually explicit material exposure class on sexually violent behavior among first-year university men in Hanoi, Vietnam, control group only (n=374)

**Outcome**	**uPR**	**95% CI**	**aPR**	**95% CI**	**ATE with IPWRA**	**95% CI**
Non-contact SV						
Class 2/3 vs. 1	4.36**	[2.64-7.20]	3.07**	[1.79-5.28]	1.19**	[1.10-1.30]
Class 3 vs. 1/2	4.03**	[2.67-6.08]	2.51**	[1.55-4.07]	1.24**	[1.09-1.41]
Any contact SV						
Class 2/3 vs. 1	3.34**	[2.12-5.27]	2.65**	[1.69-4.15]	1.17**	[1.08-1.27]
Class 3 vs. 1/2	3.82**	[2.58-5.67]	2.83**	[1.87-4.28]	1.25**	[1.11-1.40]
Contact SV: physical tactics						
Class 2/3 vs. 1	4.25**	[2.19-8.28]	3.32**	[1.67-6.62]	1.11**	[1.03-1.19]
Class 3 vs. 1/2	6.35**	[3.54-11.38]	4.68**	[2.41-9.09]	1.21**	[1.08-1.36]
Contact SV: non-physical tactics						
Class 2/3 vs. 1	3.19**	[1.99-5.12]	2.39**	[1.50-3.81]	1.13**	[1.04-1.22]
Class 3 vs. 1/2	3.66**	[2.41-5.56]	2.55**	[1.66-3.92]	1.18**	[1.09-1.18]

*Significant at <0.05; **Significant at <0.01; uPR=unadjusted prevalence ratio; aPR=adjusted prevalence ratio; ATE with IPRWA=average treatment effect with inverse probability weighted regression adjustment.
