# Supplementary material for: Effects of exposure to sexually explicit material on sexually violent behavior among first-year university men in Vietnam
Source: PLoS One. 2022 Sep 27;17(9):e0275246. doi: 10.1371/journal.pone.0275246 (PMC9514651; doi:10.1371/journal.pone.0275246)
Supplement: S2 Table — (PDF) [file pone.0275246.s002.pdf]

S2 Table. Effect of categorical exposure sexually explicit material on sexually violent behavior among first-year university men in Hanoi, Vietnam (n=739)

Outcome and level of exposure (0-3)	uPR	95% Confidence Interval	aPR	95% Confidence Interval	ATE with IPWRA	95% Confidence Interval
Non-contact SV						
1 vs. 0	1.47	[0.62-3.47]	1.29	[0.57-2.96]	1.00	[0.92-1.08]
2 vs. 0	1.10	[0.46-2.65]	0.89	[0.39-2.06]	0.97	[0.91-1.05]
3 vs. 0	4.04**	[1.93-8.45]	2.52**	[1.23-5.18]	1.12**	[1.03-1.21]
Any contact SV						
1 vs. 0	2.17	[0.90-5.24]	2.07	[0.90-4.77]	1.08	[0.99-1.17]
2 vs. 0	1.65	[0.68-4.04]	1.48	[0.63-3.48]	1.02	[0.95-1.11]
3 vs. 0	4.43**	[1.99-9.86]	3.12**	[1.43-6.85]	1.14**	[1.05-1.23]
Contact SV: physical tactics						
1 vs. 0	1.71	[0.55-5.31]	1.61	[0.54-4.77]	0.91	[0.46-1.83]
2 vs. 0	1.38	[0.44-4.29]	1.18	[0.39-3.55]	0.87	[0.43-1.75]
3 vs. 0	3.87**	[1.43-10.50]	2.64	[0.97-7.17]	0.92	[0.46-1.85]
Contact SV: non-physical tactics						
1 vs. 0	1.83	[0.74-4.51]	1.70	[0.73-3.98]	1.05	[0.96-1.13]
2 vs. 0	1.65	[0.68-4.04]	1.44	[0.61-3.41]	1.02	[0.95-1.11]
3 vs. 0	4.21**	[1.89-9.38]	2.88*	[1.32-6.29]	1.12**	[1.04-1.21]

*Significant at <0.05; **Significant at <0.01; uPR=unadjusted prevalence ratio; aPR=adjusted prevalence ratio; ATE with IPRWA=average treatment effect with inverse probability weighted regression adjustment.

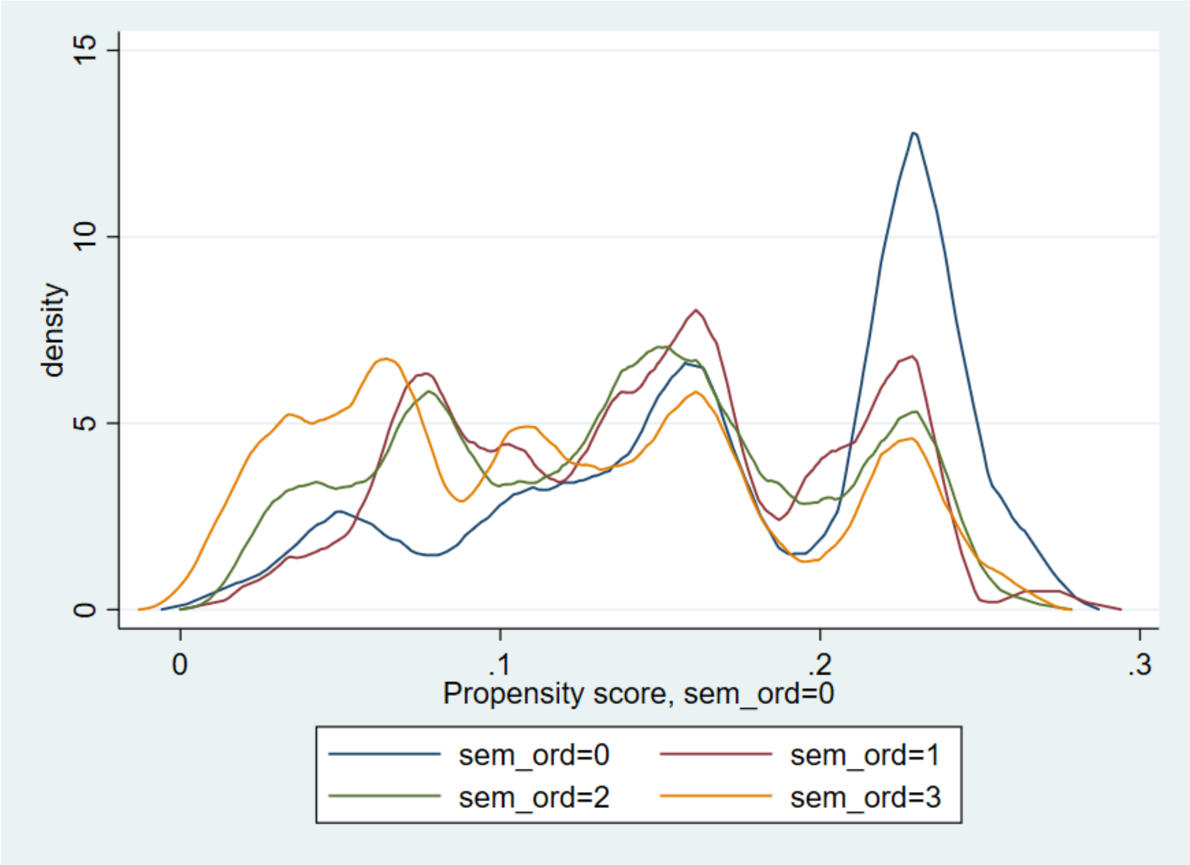

density
15
10
5
0
0
.1
.2
.3
Propensity score, sem_ord=0
sem_ord=0
sem_ord=1
sem_ord=2
sem_ord=3
