# Supplementary material for: Effects of exposure to sexually explicit material on sexually violent behavior among first-year university men in Vietnam
Source: PLoS One. 2022 Sep 27;17(9):e0275246. doi: 10.1371/journal.pone.0275246 (PMC9514651; doi:10.1371/journal.pone.0275246)

S3 Table. Over-identification and endogeneity tests for models of effect of exposure to sexually explicit material on sexual violence perpetration among first-year university men in Hanoi, Vietnam (n=735)

**Outcome**	**Over-identification**			**Endogeneity**		
Non-contact SV	χ2	df	p	χ2	df	p
0 vs. 1/2/3	13.57	9	0.1386	1.90	2	0.3684
0/1 vs. 2/3	4.41	9	0.8827	0.35	2	0.8393
0/1/2 vs. 3	7.83	9	0.5518	0.25	2	0.8838
Any contact SV	χ2	df	P	χ2	df	P
0 vs. 1/2/3	13.57	9	0.1386	7.91	2	0.0192
0/1 vs. 2/3	4.41	9	0.8827	9.58	2	0.0083
0/1/2 vs. 3	7.83	9	0.5518	10.93	2	0.0042
Contact SV: physical tactics	χ2	df	p	χ2	df	P
0 vs. 1/2/3	13.57	9	0.1386	4.95	2	0.0843
0/1 vs. 2/3	4.41	9	0.8827	7.26	2	0.0265
0/1/2 vs. 3	7.83	9	0.5518	7.04	2	0.0297
Contact SV: non-physical tactics	χ2	df	p	χ2	df	P
0 vs. 1/2/3	13.57	9	0.1386	9.61	2	0.0082
0/1 vs. 2/3	4.41	9	0.8827	9.01	2	0.0110
0/1/2 vs. 3	7.83	9	0.5518	10.29	2	0.0058

Exposure categories were as follows: 0=no exposure; 1=nudity or text only; 2=SEM with sexual acts; 3=SEM with violent sexual acts.

Overlap diagram for exposure to any sexually explicit material vs. none)

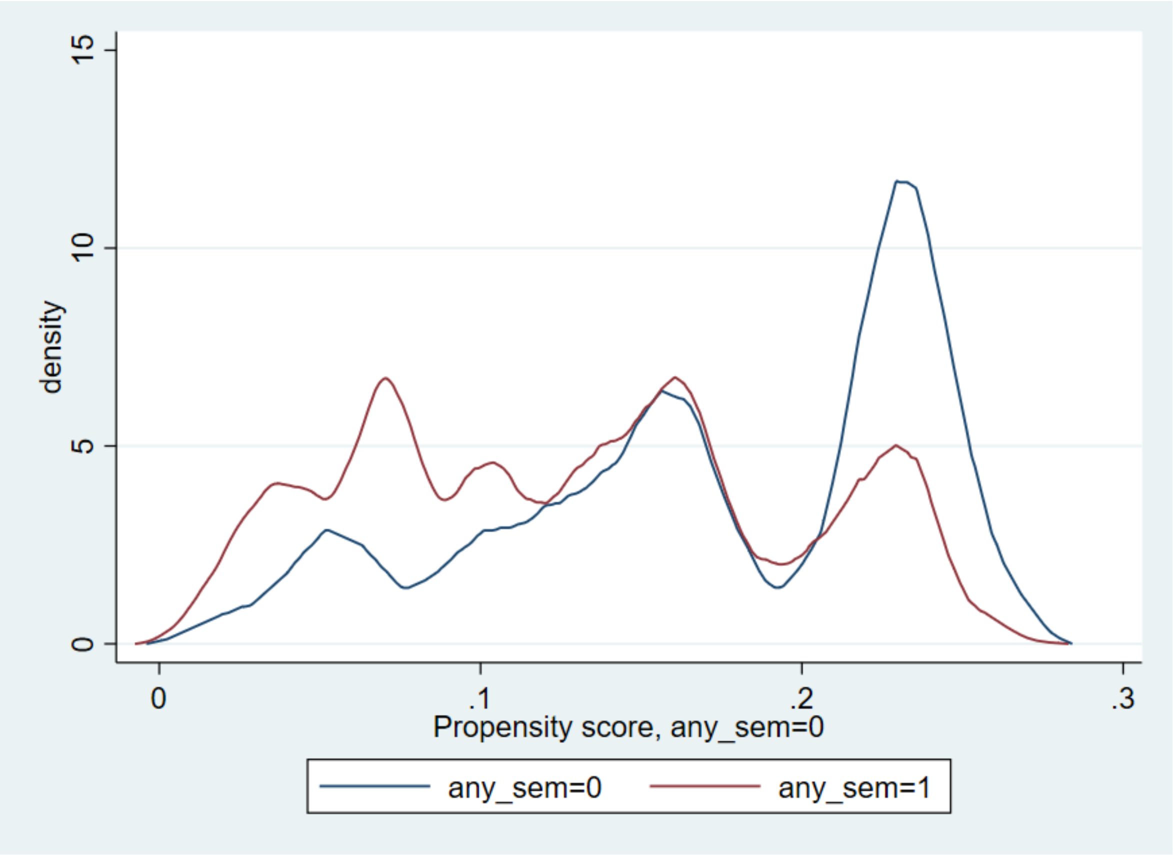

Overlap diagram for exposure to any sexual acts vs. other

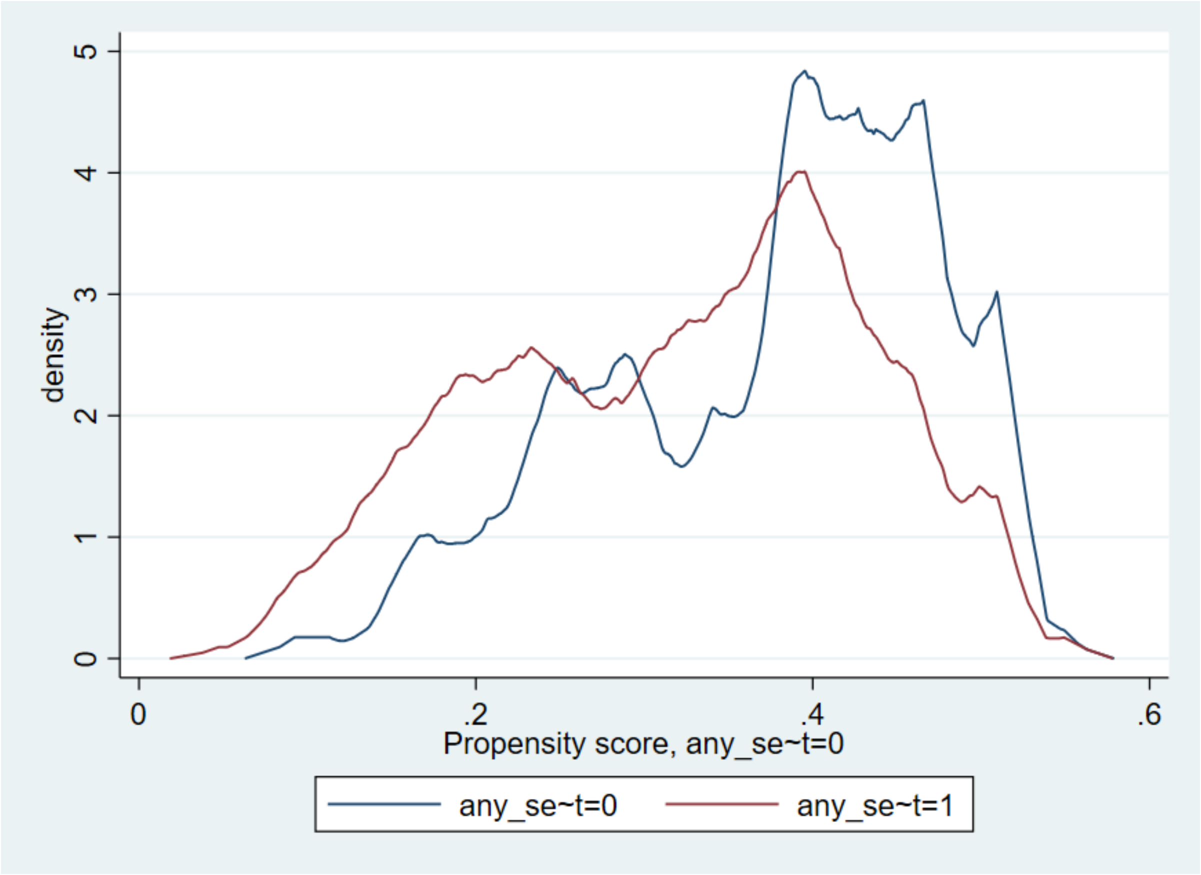

Overlap diagram for exposure to violent sexual acts vs. none

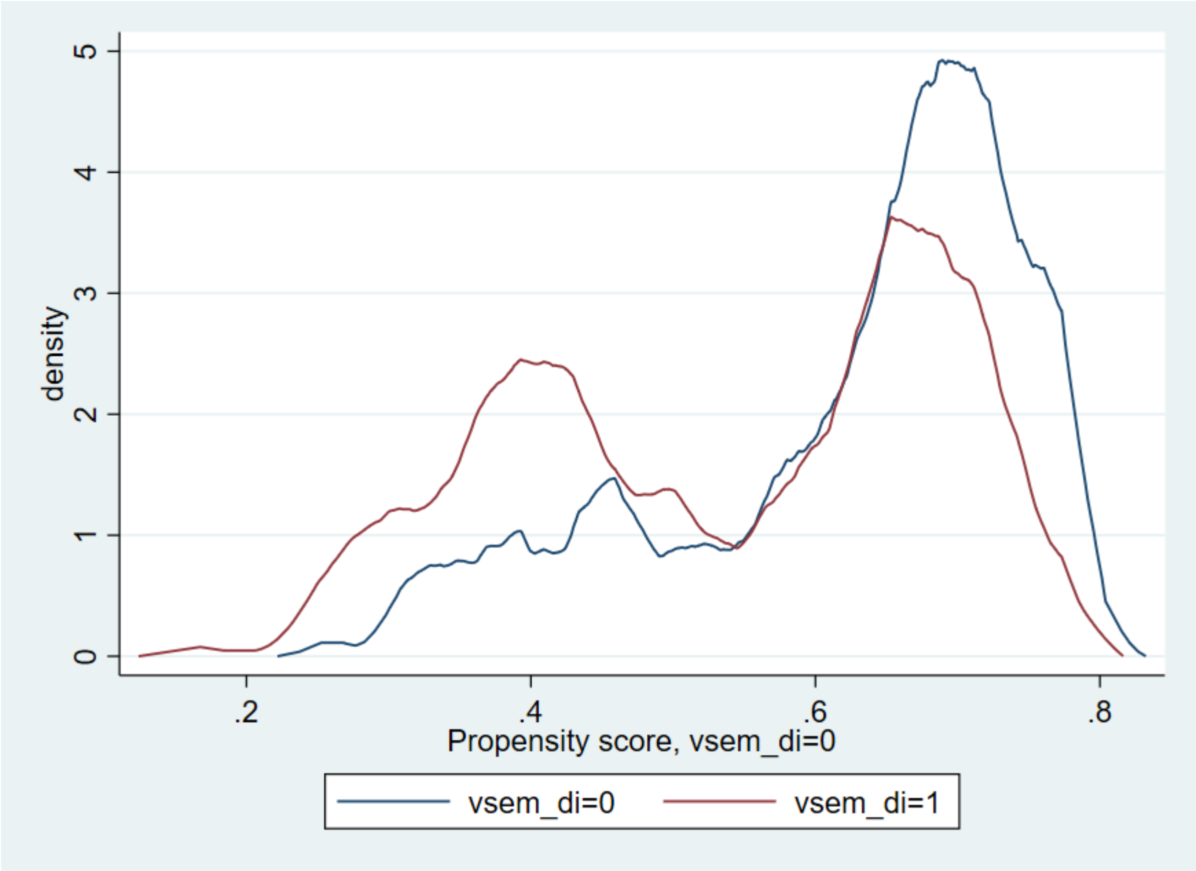

Supplement: S3 Table — (PDF) [file pone.0275246.s003.pdf]
