# Supplementary material for: Effects of exposure to sexually explicit material on sexually violent behavior among first-year university men in Vietnam
Source: PLoS One. 2022 Sep 27;17(9):e0275246. doi: 10.1371/journal.pone.0275246 (PMC9514651; doi:10.1371/journal.pone.0275246)
Supplement: S4 Table — (PDF) [file pone.0275246.s004.pdf]

S4 Table. Fit statistics for two to five latent classes of exposure to violent sexually explicit material

**Model**	**2 Classes**	**3 Classes**	**4 Classes**	**5 Classes**
Sample size	737	737	737	737
Number of free parameters	33	50	67	84
Log likelihood	-2137·192	-1773·850	-1642·374	-1579·329
AIC	4340·383	3647·69	3418·747	3326·658
BIC	4492·269	3877·82	3727·121	3713·275
Adjusted BIC	4387·483	3719·053	3514·373	3446·546
Entropy	0·971	0·979	0·982	0·984
Average Latent Class Probabilities for Most Likely Latent Class Membership	0·974; 1·000	0·993; 0·987; 0·993	0·997; 0·983; 0·994; 0·988	0·994; 0·990; 0·994; 0·983; 0·981
Vuong-Lo-Mendell-Rubin Likelihood Ratio Test (2 Times the Loglikelihood Difference)	1942	726	262	127
P-value	0	0·2377	0·0031	0·6966
