# Supplementary material for: Effects of exposure to sexually explicit material on sexually violent behavior among first-year university men in Vietnam
Source: PLoS One. 2022 Sep 27;17(9):e0275246. doi: 10.1371/journal.pone.0275246 (PMC9514651; doi:10.1371/journal.pone.0275246)

S5 Table. Over-identification and endogeneity tests for models of frequency of exposure to violent SEM among first-year university men in Hanoi, Vietnam (n=733)

Outcome	Over-identification			Endogeneity		
Non-contact SV	$\chi 2$	df	p	$\chi 2$	df	p
Class 2/3 vs. 1	2.82	9	0.9710	2.89	2	0.2361
Class 3 vs. 1/2	12.39	9	0.1922	0.08	2	0.9615
Any contact SV	$\chi 2$	df	P	$\chi 2$	df	P
Class 2/3 vs. 1	2.82	9	0.9710	7.18	2	0.0276
Class 3 vs. 1/2	12.39	9	0.1922	5.83	2	0.0541
Contact SV: physical tactics	$\chi 2$	df	p	$\chi 2$	df	P
Class 2/3 vs. 1	2.82	9	0.9710	4.84	2	0.0889
Class 3 vs. 1/2	12.39	9	0.1922	4.70	2	0.0953
Contact SV: non-physical tactics	$\chi 2$	df	p	$\chi 2$	df	P
Class 2/3 vs. 1	2.82	9	0.9710	7.15	2	0.0280
Class 3 vs. 1/2	12.39	9	0.1922	5.85	2	0.0536

Frequency classes are as follows: 1=unexposed to violent SEM; 2=exposed less than monthly; 3=exposed ≥1 time per month.

Overlap diagram for frequency of exposure to violent sexually material (3 latent classes)

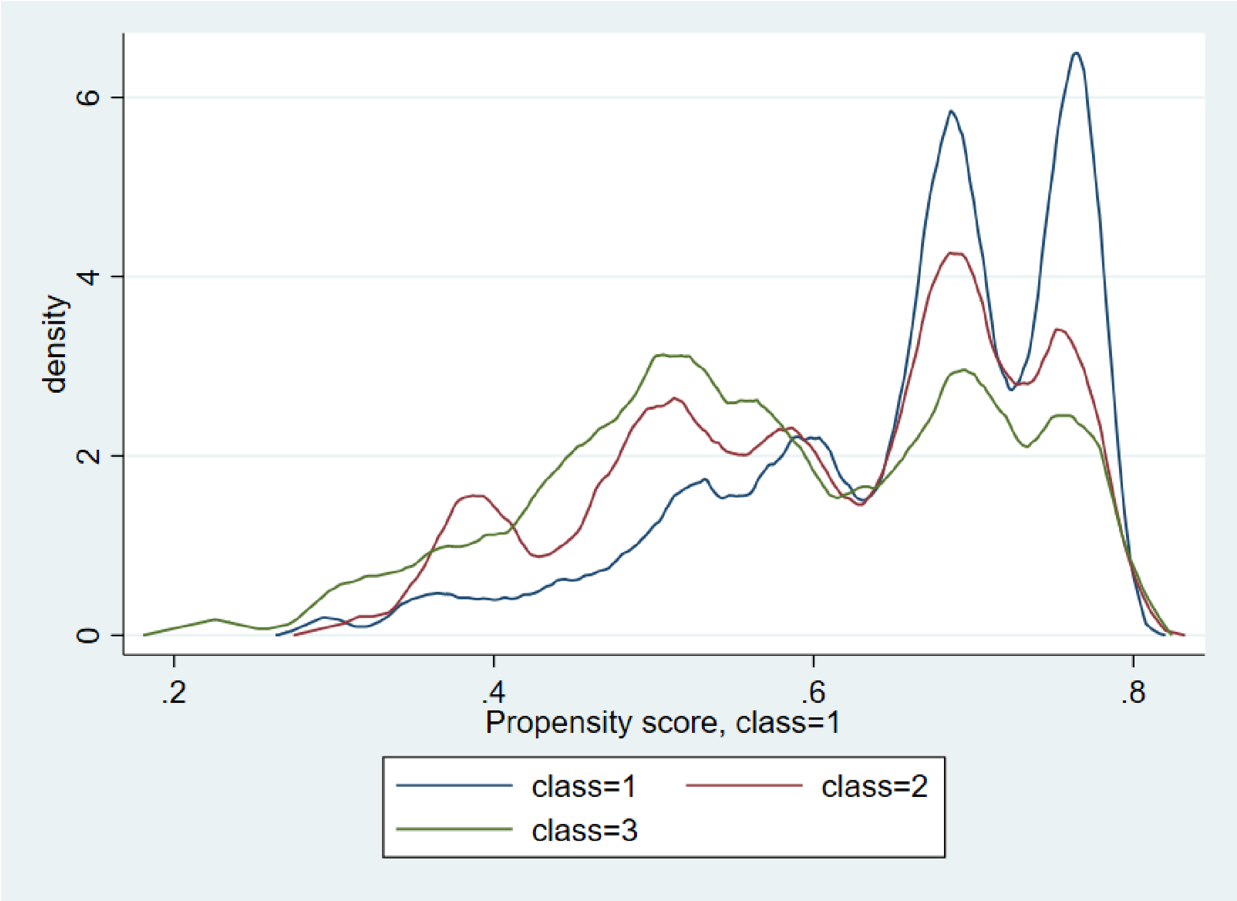

Overlap diagram for frequency of exposure to violent sexually material (frequent class vs. other)

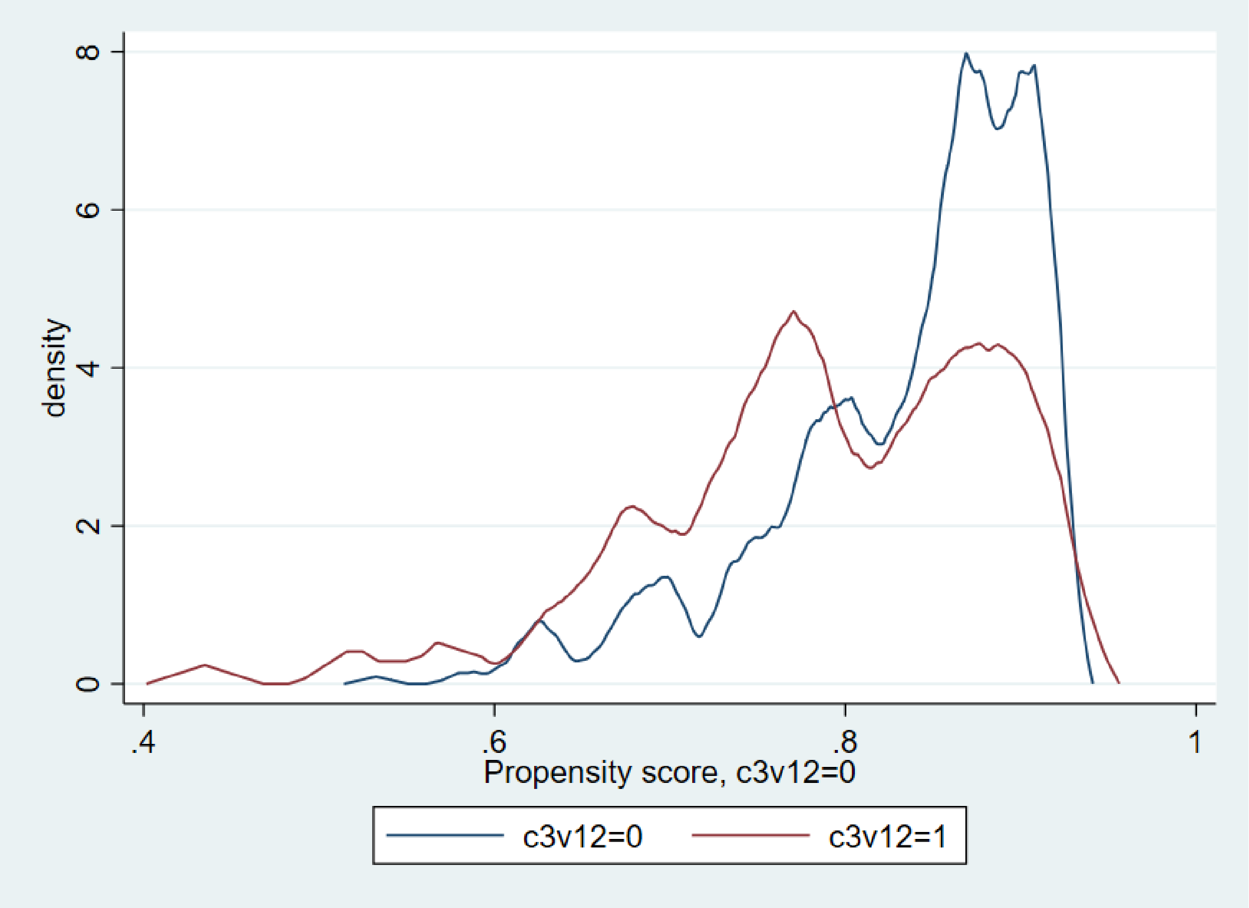

Overlap diagram for frequency of exposure to violent sexually material (frequent/infrequent vs. none)

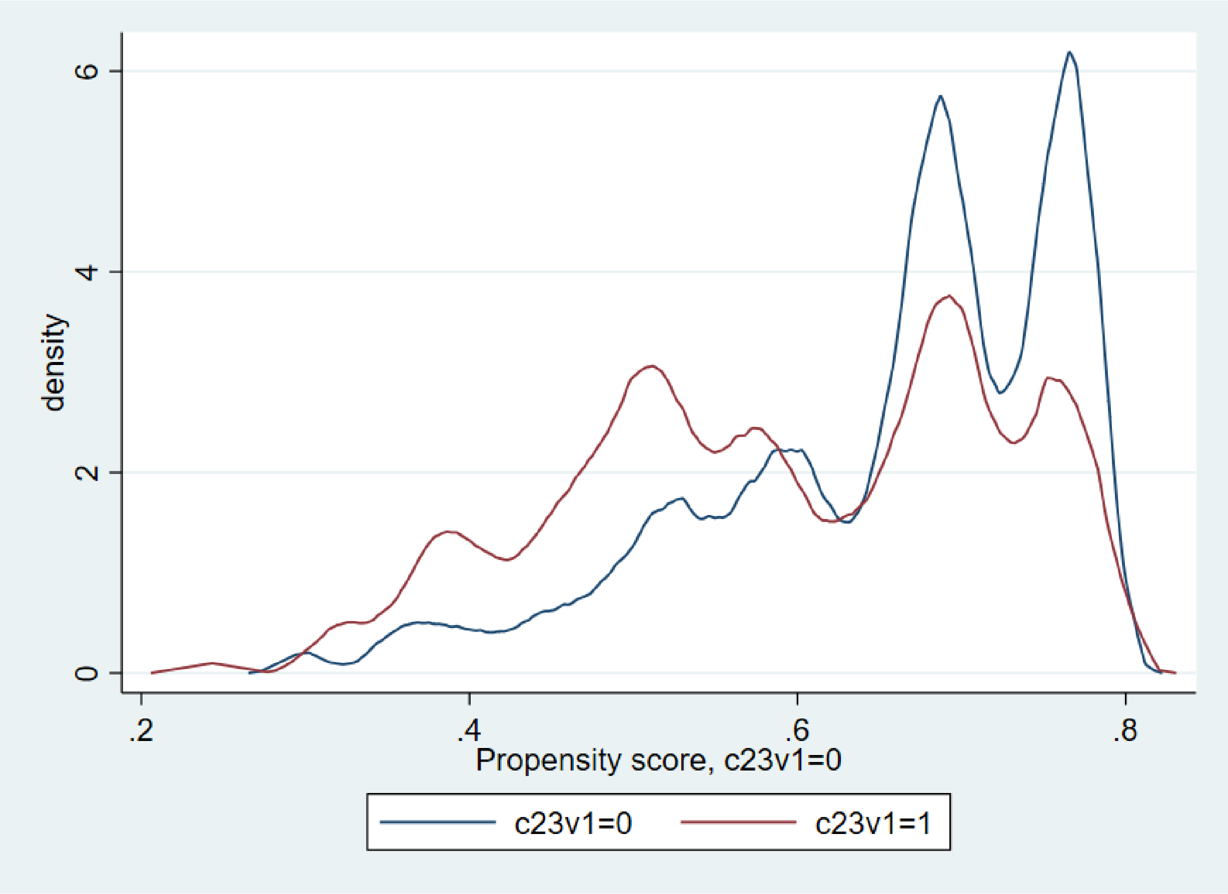

Supplement: S5 Table — (PDF) [file pone.0275246.s005.pdf]
